# Fictive Scratching Patterns in Brain Cortex-Ablated, Midcollicular Decerebrate, and Spinal Cats

**DOI:** 10.3389/fncir.2020.00001

**Published:** 2020-02-27

**Authors:** Irene Guadalupe Aguilar Garcia, Judith Marcela Dueñas-Jiménez, Luis Castillo, Laura Paulina Osuna-Carrasco, Braniff De La Torre Valdovinos, Rolando Castañeda-Arellano, Jose Roberto López-Ruiz, Carmen Toro-Castillo, Mario Treviño, Gerardo Mendizabal-Ruiz, Sergio Horacio Duenas-Jimenez

**Affiliations:** ^1^Departamento de Biología Molecular y Genómica, CUCS, Universidad de Guadalajara, Guadalajara, Mexico; ^2^Departamento de Fisiología, CUCS, Universidad de Guadalajara, Guadalajara, Mexico; ^3^Centro Básico, Universidad de Aguascalientes, Aguascalientes, Mexico; ^4^Departmento de Electrónica y Computación, CUCEI, Universidad de Guadalajara, Guadalajara, Mexico; ^5^Departmento de Ciencias Biomédicas, CUTonala, Universidad de Guadalajara, Guadalajara, Mexico; ^6^Departmento de Neurociencias, CUCS, Universidad de Guadalajara, Guadalajara, Mexico; ^7^Laboratorio de Plasticidad Cortical y Aprendizaje Perceptual, Instituto de Neurociencias, Universidad de Guadalajara, Guadalajara, Mexico

**Keywords:** fictive scratching, motor patterns, decerebrate cat, spinal cat, monosynaptic reflex

## Abstract

**Background**: The spinal cord’s central pattern generators (CPGs) have been explained by the symmetrical half-center hypothesis, the bursts generator, computational models, and more recently by connectome circuits. Asymmetrical models, at odds with the half-center paradigm, are composed of extensor and flexor CPG modules. Other models include not only flexor and extensor motoneurons but also motoneuron pools controlling biarticular muscles. It is unknown whether a preferred model can explain some particularities that fictive scratching (FS) in the cat presents. The first aim of this study was to investigate FS patterns considering the aiming and the rhythmic periods, and second, to examine the effects of serotonin (5HT) on and segmental inputs to FS.

**Methods**: The experiments were carried out first in brain cortex-ablated cats (BCAC), then spinalized (SC), and for the midcollicular (MCC) preparation. Subjects were immobilized and the peripheral nerves were used to elicit the Monosynaptic reflex (MR), to modify the scratching patterns and for electroneurogram recordings.

**Results**: In BCAC, FS was produced by pinna stimulation and, in some cases, by serotonin. The scratching aiming phase (AP) initiates with the activation of either flexor or extensor motoneurons. Serotonin application during the AP produced simultaneous extensor and flexor bursts. Furthermore, WAY 100635 (5HT1A antagonist) produced a brief burst in the tibialis anterior (TA) nerve, followed by a reduction in its electroneurogram (ENG), while the soleus ENG remained silent. In SC, rhythmic phase (RP) activity was recorded in the soleus motoneurons. Serotonin or WAY produced FS bouts. The electrical stimulation of Ia afferent fibers produced heteronymous MRes waxing and waning during the scratch cycle. In MCC, FS began with flexor activity. Electrical stimulation of either deep peroneus (DP) or superficial peroneus (SP) nerves increased the duration of the TA electroneurogram. Medial gastrocnemius (MG) stretching or MG nerve electrical stimulation produced a reduction in the TA electroneurogram and an initial MG extensor burst. MRes waxed and waned during the scratch cycle.

**Conclusion**: Descending pathways and segmental afferent fibers, as well as 5-HT and WAY, can change the FS pattern. To our understanding, the half-center hypothesis is the most suitable for explaining the AP in MCC.

## Introduction

The central pattern generators (CPGs) in the spinal cord were first studied by Brown and Sherrington (1911), who proposed the symmetrical half-center hypothesis. The burst generator concept emerged many decades later (Anderssson and Grillner, 1981) and was followed by the development of computational models (Shevtsova and Rybak, 2016; Ausborn et al., 2018, 2019). Recently, connectome circuits have been reported in genetically identified interneurons (for a comprehensive review, see Rybak et al., 2015).

Asymmetrical models are conformed by extensor and flexor CPG modules working independently but are at odds with the half-center paradigm (Hägglund et al., 2013). Other concepts that include not only flexor and extensor motoneurons but also motoneuron pools controlling bi-articular muscles are being developed (Barardi et al., 2014). There are other models based on a two-layer longitudinal architecture of the CPG that propose a theoretical circuit that reproduces the sinusoidal shape of the dorsal cord potentials (Pérez et al., 2009). Interneurons within extensor module circuits that possess rhythmic properties could be sufficient to perform different rhythmic motor tasks (Frigon and Gossard, 2010).

During fictive locomotion (FL) or fictive scratching (FS), flexor and extensor commissural interneurons share both motor tasks (Trejo et al., 2015). Moreover, the spinal cord CPG seems to be composed of neural elements in two levels: one generates the rhythm and the other produces several motor patterns in the hindlimb extensor and flexor motoneurons (Rybak et al., 2006a,b). These levels are related to the interneurons that generate the motor pattern such as the aiming phase (AP) and rhythmic phase (RP) during scratching. The segmental peripheral and descending inputs could affect the scratching motor task during the two different phases. Descending serotoninergic inputs increase the excitability of the CPG’s spinal extensor motoneurons. These serotoninergic pathways could originate either from brainstem neurons giving rise to the descending tracts (Jordan et al., 2008) or from propriospinal interneurons (Cabaj et al., 2017). Some studies have reported that monoamines are strong modulators and/or activators of the spinal locomotor networks (Noga et al., 2009, 2017), although they are not necessary to evoke locomotion (Steeves et al., 1980). Sustained monoamine release activates receptors in neurons within the spinal cord, suggesting sustained extrasynaptic transmission (Noga et al., 2017). FS activity is also facilitated by topical d-tubocurarine spinal cord application induced by pinna stimulation (Domer and Feldberg, 1960). Numerous studies of spinal cord locomotor circuits’ pharmacological modulation in decerebrated or in spinal cord-injured cats have provided insight into the roles of monoamines in posture and stepping (Barbeau and Rossignol, 1991; Rossignol et al., 1998, 2001; Brustein and Rossignol, 1999). Modulation of the monoaminergic system in cats, rats, and turtles has allowed the observation of motoneuron excitability regardless of the use of typical facilitation mechanisms to affect membrane potential (Hornby et al., 2002; Fedirchuk and Dai, 2004; Gilmore and Fedirchuk, 2004). It is important to emphasize that the scratching phenomenon in fictive motor preparations has not been deeply investigated to establish the afferent and descendent pathway producing FS in cats. A relevant work was made in a mice model, which exhibits the pathways related to itch and their relationship to periaqueductal gray glutamatergic neurons (Gao et al., 2019).

In brain cortex-ablated cats (BCAC), the AP in FS could initiate with either extensor or flexor tonic activity and is followed by the RP (Duenas-Jimenez et al., 2017). Interestingly, the AP in most midcollicular cats (MCC) starts with flexor tonic activity (Berkinblit et al., 1978). In BCAC, it remains unclear whether extensor motoneurons are inhibited during the AP as postulated by the half-center hypothesis. Therefore, one of the purposes of this work was to clarify whether the activation of the extensor half center during the AP in BCAC inhibits the flexor one, and vice versa, as the half center hypothesis predicts. Balanced excitation–inhibition and irregular firing modified by serotonin (5HT) are fundamental motifs in certain spinal network functions (Berg et al., 2007; Guzulaitis et al., 2016). It is also known that increased synaptic fluctuations in membrane potential, irregular firing, and increased conductance occur in spinal motoneurons during scratching network activity. Serotonin produces irregular firing in turtle motoneurons and modifies the actions between flexor and extensor half centers; however, its effect in cats is not known. Motoneuron activation is a general feature of spinal motor network activity (Guzulaitis et al., 2016); thus, we can conjecture that motoneurons that do not fire during FS could be driven by a different spinal cord oscillator. In the BCAC, it is unknown whether a different subthreshold rhythm could be elicited during FS in silent soleus (SOL) motoneurons.

Several recent studies have assessed the relationship between brainstem and spinal cord motoneurons to evaluate the neural circuits involved in the rhythm generator for FS. Therefore, an additional goal was to evaluate the effect of injecting 5HT in the caudal brainstem on the FS patterns generation in BCAC that were not induced by d-tubocurarine and pinna stimulation. The 5HT1A receptor is implicated in other rhythmic activities such as swimming in *Xenopus laevis* (Wedderburn and Sillar, 1994) and in the respiratory rhythm of mice. Therefore, the 1A receptor antagonist drug WAY 100635 can be used to study 5HT effects on FS patterns (Dhingra et al., 2016). Systemic and focal 5HT1a receptor blockade with WAY100635 increased the occurrence of spontaneous apneas and rhythm variability. 5HT1aR-dependent synaptic transmission in the dorsolateral pontine nucleus modulates respiratory network stability through 5HT1a receptors, regulating the frequency of spontaneous apneas; hence, there is intrinsic respiratory variability in response to extrinsic chemosensory perturbations (Dhingra et al., 2016).

In BCAC, the heteronymous monosynaptic reflex (HeMR) produced by Ia afferent fibers acting in motoneurons of the same joint has been previously analyzed (Duenas-Jimenez et al., 2017). However, these reflexes were not fully analyzed among the Ia afferent fibers and motoneurons innervating the hindlimb muscles acting in different joints. Here, we studied whether Ia afferent fibers are producing HeMR in the semitendinosus (ST), lateral gastrocnemius (LG), and flexor hallucis longus (FHL) motoneurons by posterior biceps (PB) stimulation and whether medial gastrocnemius (MG) electrical stimulation of Ia afferent fibers evoke HeMR in FHL motoneurons. The effects of electrical stimulation of deep peroneus (DP), superficial peroneus (SP), and MG nerves in MCC were also evaluated to find support for either the half center or the asymmetrical hypothesis.

## Materials and Methods

### General Procedures

All procedures were performed in accordance with the ethical guidelines of the Mexican Official Norm (NOM-062-ZOO-1999) and the National Institutes of Health Guide NIH Publication No. 8023 (1996) for the Care and Use of Laboratory Animals. All experimental experiments in 39 cats produced the total data set (preparations and protocols listed in Table 1). Protocols were approved by the Institutional Animal Care and Use Committee (IACUC). Some of the data presented come from experiments previously made in 32 cats for answering other scientific questions. However, this dataset optimizes the scientific output of every animal experiment. Animals were raised and housed in separate cages.

**Table 1:** Treatment and stimulation applied in the different cats’ preparations for ENG recordings during FS.

Preparation	Treatment	NC	NE	Stimulation
BCAC-SC	-	32		-
BCAC	d-tubocurarine	4	6	-
BCAC	d-tubocurarine	5	11	Pinna + PB/MG nerves
BCAC	BS 5-HT	2	3	Pinna
BCAC	IS 5-HT	21*	45	Pinna
BCAC	IS WAY	21*	27	Pinna
SC	IS 5-HT	21*	40	Pinna
SC	IS WAY	21*	9	Pinna
MCC	-	7		Pinna + GS muscle stretching
MCC	-	7*		Pinna + MG stimulation
MCC	-	7*		Pinna + SP stimulation
MCC	-	7*		Pinna + DP stimulation

*BCAC-SC, brain cortex-ablated cats, later spinalized; BS: brainstem; DP: deep peroneus nerve; ENG: electroneurogram; FS: fictive scratching; 5HT: serotonin; IS: intra-spinal cord injection; MCC: midcollicular cats; MG, medial gastrocnemius; NC: number of cats used in the experiment; NE: number of episodes; PB: posterior biceps; SC: spinal cord; SP: superficial peroneus. The asterisk indicates the same subjects as BCAC-SC or MCC*.

Immobilized BCAC with a mass of 3–3.5 kg each were used to study FS. Brain cortex and suprathalamic structure ablation, as well as damage in some thalamic nuclei, was performed under ketamine (20 mg/kg) and brevital (20–40 mg/kg) as previously described (López Ruiz et al., 2017). For SC, the spinal cord was cut at C1–C2 segments. Some were injected with 7 μM 5HT solution (0.5 ml at 2 μl/min) in the spinal cord during brain cortex-ablated status or in the same cats after spinalization; other cats were microinjected in the spinal cord with WAY-100635 (N-[2-[4-(2-methoxyphenyl)-1-piperazinyl] ethyl]-N-(2-pyridyl) cyclohexanecarboxamide, 0.2–5 nM throughout the course of 5–20 min) during brain cortex-ablated status and 3–4 h after spinalization preparation. Some cats were injected with 5HT in the brainstem, 1 mm down the obex to evoke FS (Table 1). In MCC, following a craniotomy, the cortex was removed, and all tissue rostral to the inferior colliculi and mammillary bodies was removed. At this point, the animals were considered painless, so anesthesia was discontinued. All cats were paralyzed with pancuronium bromide injections (1 mg/kg) administered through the radial veins every 60 min until the end of the experiment. Paralysis is required for the study of centrally generated FS and the effects of specific sensory inputs, so the animals were artificially ventilated (sustained expiratory CO_2_ level from 4% to 5%) throughout the experiment. A lethal injection of pentobarbital anesthetic was administered through the jugular vein at the end of the experiment.

### FS

FS in immobilized BCAC, MCC, and SC was evoked by light and continuous pressure in the pinna after topical d-tubocurarine application (0.01–0.025%) in C1–C2 spinal cord segments (Domer and Feldberg, 1960). In two cases, 5HT was injected in brainstem to achieve FS; in four other cats, FS appeared after local d-tubocurarine application.

### ENG Recordings

ENG data acquisition and analysis from two to nine nerves was performed using AxoScope 7 software. The signal was amplified in differential alternating current. The sampling rates per channel varied from 16 to 2 kHz depending on the number of simultaneous recorded channels. FS episodes were divided into tonic AP and RP with extensor and flexor activity constituting a scratching cycle.

In MCC, the MG muscle was stretched 3–6 mm by applying force to the posterior surface of the calcaneus (calcaneus-tibial, angle 50–90°). In these experiments, the MG and LG nerves and spinal roots were kept intact except for a thin MG filament, which was dissected, sectioned, and used for recording. The DP, SP, or MG nerves were electrically stimulated with single shocks; the threshold of the most excitable nerve was determined by recording afferent volleys in the L7 or S1 using a ball electrode with a reference lead placed in the skin or back muscle.

Electrical high frequency (30–300 Hz) and variable duration were also used. Monosynaptic reflex (MR) discharges were elicited in the MG and flexor tibialis anterior (TA) nerves by electrical stimulation at 1.2–1.5 times the threshold for the most excitable fibers of the L7 or S1 dorsal roots.

### HeMRs in BCAC

The time of each scratch cycle was normalized and divided in 10 bins to increase the temporal resolution of the statistical analysis (Alaburda, 2005). The means and standard deviations (SDs) of all the peak-to-peak HeMR amplitudes corresponding to each bin of the pre- and bursting period were computed and evaluated for statistical differences using one-way analysis of variance (alpha = 0.05). An adjustment was made for the degree of freedom (Bonferroni corrections for multiple comparisons, alpha = 0.05, *n* = 10 (Welch and Ting, 2009). The obtained HeMR means (circles) and SDs (vertical lines) prior to scratching are shown in Figures 3A–D. All scratching cycles that did not differ >15% from the mean cycle duration were plotted and indicated under the abscissa; the durations of the cycle phases are also shown (extensor: E and flexor: F).

**Figure 1:**
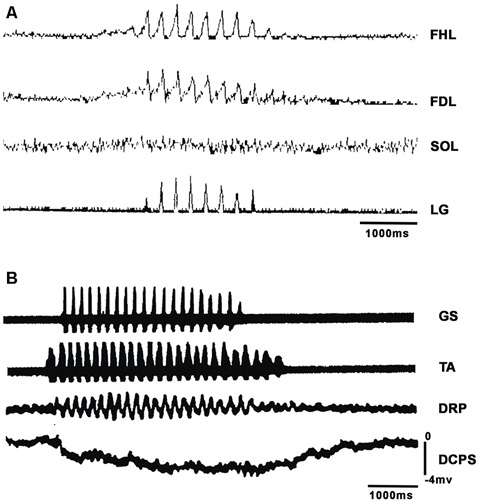
Short episodes of fictive scratching (FS) appeared after d-tubocurarine application with two characteristics: absence of SOL electroneurographic activity and DC shifts in spinal cord intermediate nucleus. **(A)** Traces: flexor hallucis longus (FHL), lateral gastrocnemius (LG), flexor digitorum longus (FDL) and soleus (SOL). The ENGs were integrated and rectified. **(B)** DC potential during FS scratching. Traces: first, gastrocnemius soleus (GS); second, tibial anterior (TA). Traces 1 and 2 are raw electroneurograms. Third trace: dorsal root potential (DRP), fourth trace: DC potential shift.

**Figure 2:**
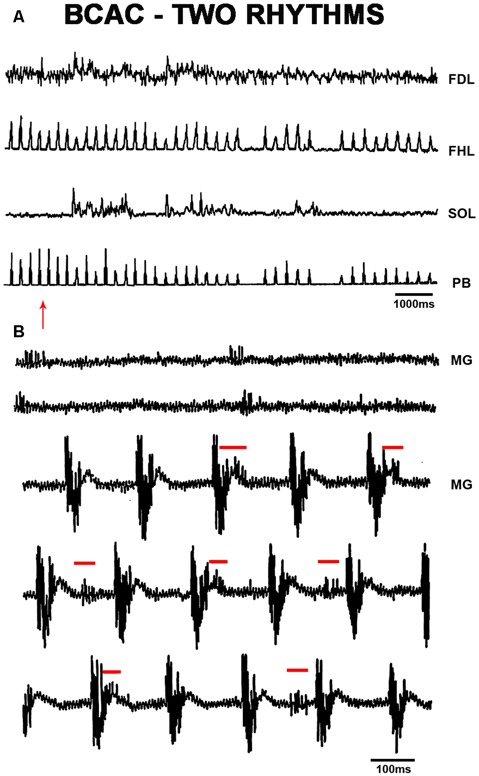
**(A)** FS produced by pinna stimulation and bursting activity with a slower frequency in SOL ENG; FS was rectified and integrated ENG in FHL, FDL and SOL ENG exhibits a slow frequency rhythm produced by FP mechanical stimulation. Traces as indicated. **(B)** Slow frequency spontaneous rhythmic activity in the MG ENG. After pinna stimulation, slow frequency rhythmic activity concurred with FS frequency in the MG ENG. The red arrow indicates FP stimulation.

**Figure 3:**
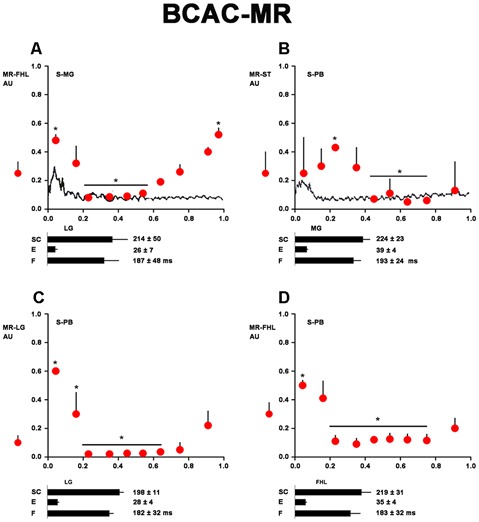
Heteronymous Monosynaptic reflex (MR) modulation in brain cortex-ablated cats (BCAC) during the rhythmic phase (RP) of the FS. **(A)** HeMR amplitude changes in FHL motoneurons (ordinate), produced by electrical MG nerve stimulation (S-MG) plotted in relation to the normalized FHL scratch cycle (abscissa). Upper bar below the abscissa (SC) denotes cycle duration as measured in the FHL ENG. Second and third bars below the abscissa are the extensor and flexor phase durations, respectively. The dot with the vertical line on the left of the ordinate indicates the HeMR amplitude and standard deviation (SD) before the scratching episode. **(B–D)** HeMR amplitude changes in ST, LG, and FHL motoneurons in relation to normalized ST, LG, and FHL scratch cycles, respectively. Electrical stimulation was given to the PB nerve (1.3 T). In B, ST-HeMR amplitude changes (ordinate) in relation to the ST scratch cycle (abscissa). The continuous line in panel **(B)** represents the averaged and rectified ST ENG. The extensor phase is indicated. The mean scratching cycle duration and its SD were measured in the ST ENG. Asterisk in **(A–D)** denotes *P* < 0.05. In **(C,D)**, the HeMR was recorded in LG and FHL nerves, respectively. Other indications as in **(A)**.

### Direct Current Potential in the BCAC Spinal Cord During Scratching

In cats, where FS only appeared after only d-tubocurarine applications, the DC potentials were measured by a microelectrode filled with sodium chloride (2 M) placed in the intermediate nucleus of the L6 spinal cord segment. A ball electrode was placed in the L6–L7 segment to record dorsal root potentials (DRPs) and the incoming potential evoked by PB or MG stimulation.

## Results

### FS Patterns in Ablated Cats

In BCAC, we analyzed FS in several conditions. In four BCAC subjects, FS appeared after topical application of d-tubocurarine to the spinal cord. Figure 1A shows the rectified and integrated FDL, FHL, SOL, and LG ENGs from one of these experiments. It is clear that most SOL motoneurons did not exhibit ENG activity during scratching. FHL and LG activity occurred in phase. In two of these subjects, phasic DRP and a sustained DC potential appeared during RP activity (Figure 1B). This point is very important since actual models do not consider the electrical and the companying magnetic field related to the RP. The absence of activity in most SOL motoneurons is also noteworthy, since these silent motoneurons could be used by the locomotor CPG to act in some segregated motoneurons.

In BCAC, FS episodes were observed in FHL and PB nerves following pinna stimulation, and foot pad (FP) mechanical stimulation (indicated by an arrow) produced FL in the SOL nerve, thus concurring both rhythms (Figure 2A). During spontaneous FL recorded in the MG ENG, pinna stimulation produced FS (5 Hz) concurrent with the spontaneous low-frequency rhythm (2 Hz; Figure 2B), likely indicating a possible coupling between slow and fast oscillators (Ausborn et al., 2018; Frigon and Gossard, 2010). We did not record alternating activity between TA and MG or SOL; it seems possible that bursting activity occurs in some extensor motoneurons but not rhythmic activity in flexor motoneurons of the same joint. Then, we decided to study the HeMR between Ia afferent fibers to clarify if spinal scratching generator modulates the motoneuron activity simultaneously in distal joints.

### Heteronymous Monosynaptic Reflex in BCAC During FS Produced by Pinna Stimulation

In five cats treated with d-tubocurarine, FS occurred after pinna stimulation. In these cats, we analyzed the HeMR produced in the motoneurons innervating muscles acting in different joints by stimulating the group Ia afferent fibers (Figure 3). MG stimulation produced HeMR in FHL motoneurons, waxing and waning during the scratch cycle (Figure 3A). PB electrical stimulation produced HeMR in the ST, FHL, and LG. These reflexes also waxed and waned during the scratch cycle (Figures 3B–D).

### FS Patterns in BCAC and SC With Serotonin and WAY Application

**Figure 4:**
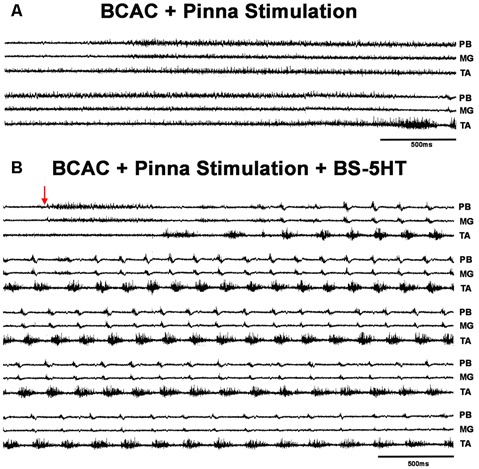
FS episode elicited by pinna stimulation plus 5HT in a BCAC. **(A)** Pinna stimulation without 5HT (traces as indicated MG, TA, and PB). **(B)** After brainstem 5HT microinjection indicated with an arrow (traces as indicated PB, MG, and TA). The traces are consecutive recordings. In **(B)**, the ENG activity before RP onset occurred in PB and MG. The red arrow indicates pinna stimulation.

**Figure 5:**
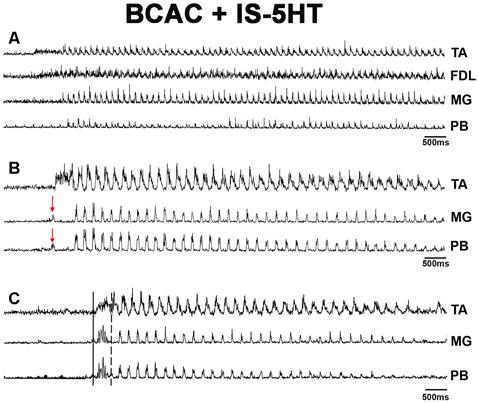
Different FS patterns in BCAC produced by pinna stimulation plus 5HT. **(A)** The AP commences with flexor activity in the TA ENG. **(B)** FS in a different cat after spinal cord 5HT microinjection. The AP commences with ENG activity in the MG and PB ENG. **(C)** FS episode showing tonic AP with simultaneous activity on flexor and extensor ENG. FS episodes **(A–C)** were recorded in three different cats. ENGs, integrated and rectified. The red arrow indicates burst activity in MG and PB, ENG before TA-ENG activity during the AP.

**Figure 6:**
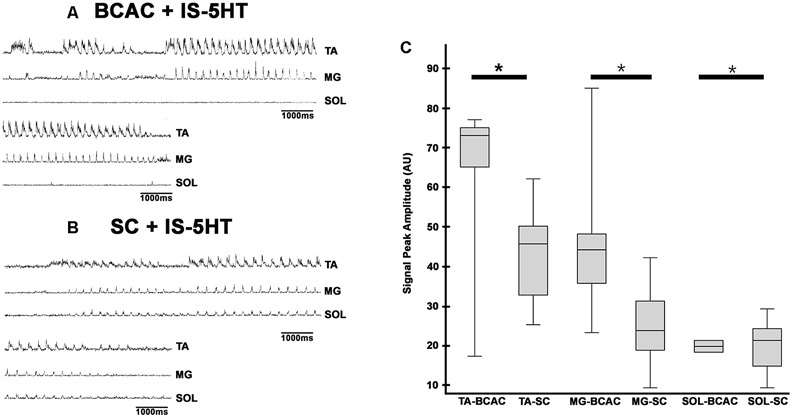
FS episode elicited by pinna stimulation in a BCAC and SC after intraspinal cord 5HT microinjection. (**A)** BCAC, traces as indicated (TA, MG, and SOL). **(B)** SC, traces as indicated (TA, MG, and SOL), note the scratching SOL ENG activity. ENGs rectified and integrated. **(C)** The graph insert shows the peak amplitude means values of TA, MG, and SOL, *P* < 0.05.

**Figure 7:**
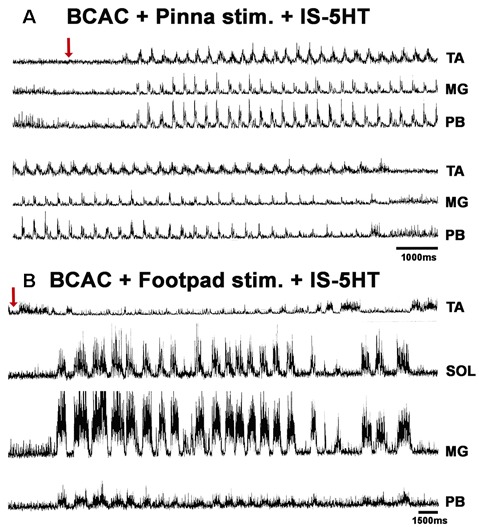
FS and fictive locomotion (FL) evoked in a BCAC by pinna stimulation and mechanical FP stimulation with 5HT applied in the spinal cord. **(A)** An episode of FS evoked by pinna stimulation in a 5HT-treated cat. The red arrow indicates pinna stimulation. **(B)** An episode of FL evoked by FP stimulation in a 5HT-treated cat. The red arrow indicates FP stimulation.

**Figure 8:**
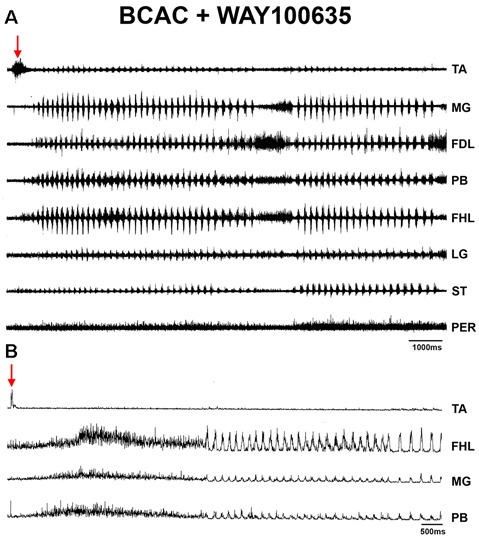
**(A)** ENGs of eight hindlimb nerves during an episode of FS evoked by pinna stimulation after WAY-100635 microinjection in the L4 spinal cord in a BCAC. Traces as indicated (TA, MG, FDL, PB, FHL, LG, ST, and PER). **(B)** FS in a different cat produced by pinna stimulation and WAY-100635 in the L5 segment in the spinal cord of a BCAC. Traces as indicated (TA, FHL, GM, and PB). Note the TA ENG activity at the onset of the FS episode, also a sustained reduction in TA ENG during the RP. The red arrows indicates pinna stimulation with a burst in the TA-ENG.

**Figure 9:**
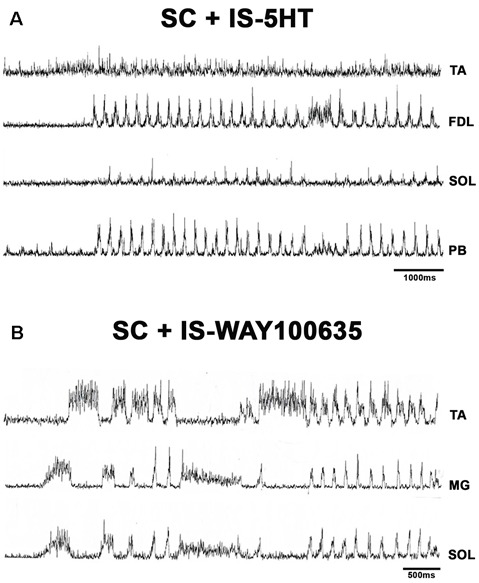
**(A)** FS in spinal cat after 5HT microinjection. Traces as indicated (TA, FDL, SOL, and PB). 5HT produces rhythmic ENG activity in the SOL nerve. **(B)** FS in an SC after microinjection of WAY-100635. Traces as indicated (TA, GM, and SOL). EMG traces were integrated and rectified.

**Figure 10:**
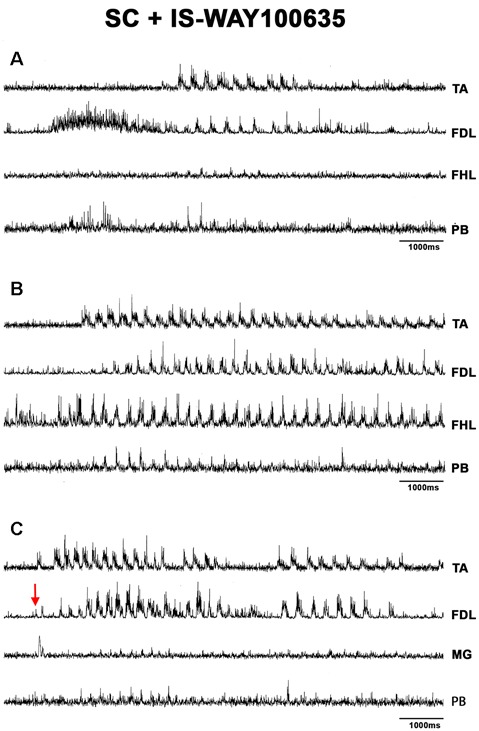
FS bouts in spinal cats produced by WAY-100635, FS commences with long duration bursts in FDL and PB ENGs **(A)**, with a burst in FHL **(B)**, or short bursts in TA, FDL, and MG nerves **(C)**. ENG amplitude varied for different bouts of FS (TA, FDL, MG, and PB). EMG traces were integrated and rectified. The red arrow indicates pinna stimulation and the FS episode begun with a short burst of activity in TA, FDL, MG nerves.

When pinna stimulation did not produce FS (Figure 4A), 5HT brainstem microinjection did (Figure 4B). In 5HT-treated cats, the AP sometimes started with flexor activity (Figure 5A) as seen in cats that were not treated with 5HT. In other subjects, the AP initiated with simultaneous flexor and extensor ENG activity (Figure 5C) or with a short burst in the extensor nerves (Figure 5B, arrows). In BCAC, the SOL ENG remained silent, but activity occurred in SC; also, there was a notable reduction in TA and MG ENG activity in SC compared with BCAC, statistically significant as shown in the graph figure (Figures 6A–C). In five cats, when pinna stimulation induced FS, FP stimulation produced FL (Figure 7). Microinjection of WAY-100635 into the spinal cord produced a FS episode after a short TA ENG burst, which was followed by a TA ENG amplitude reduction (Figure 8A). In two cases, the initial TA burst was followed by tonic activity in the extensor ENG that continued until RP onset (Figure 8B). In SC, spinal cord 5HT microinjections produced bouts of FS (Figure 9A). WAY-100635 intraspinal cord injections also elicited FS (Figure 9B) that began with activity in the extensor motoneurons, although AP patterns was variable in other scratching episodes (Figures 10A–C).

### FS Patterns and the MR in MCC

**Figure 11:**
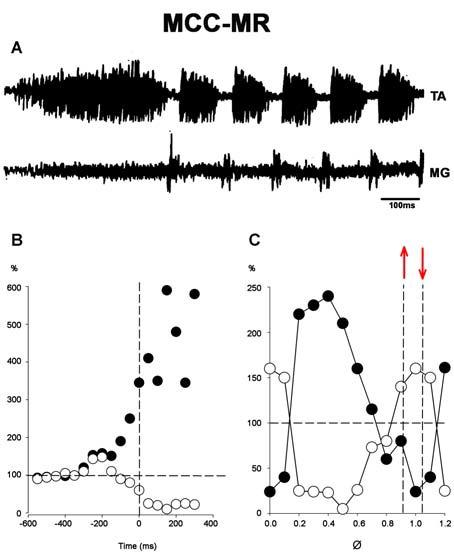
**(A)** An episode of FS in an MCC recorded in the TA and MG nerves. **(B)** Normalized amplitudes of MR responses before and during the approach period in a single experiment. AP onset is indicated by the vertical interrupted line at time zero. The reflex is produced by single electrical shocks applied to the sectioned L7 roots at variable intervals before or after the beginning of pinna stimulation. Black dots, TA; white dots, MG; MR amplitude values. **(C)** Normalized MR amplitude during the rhythmic period. 100% value indicated by a hatched line in the ordinate (note the logarithmic scale in ordinates of **C**). Abscissa normalized cycle. The values between hatched lines correspond to MG MR activity. Between red arrows indicate the extensor phase of the scratch cycle.

**Figure 12:**
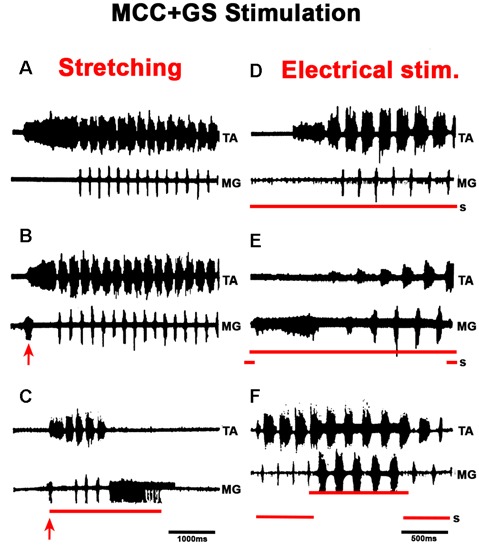
Effects produced by activation of afferent fibers from extensor muscles in the FS pattern. Nerve activity was recorded from the TA nerve and a small filament in the MG nerve. Panels **(A–C)**, **(D–F)**, two different experiments. Panels **(A,D)** are control ENGs, traces as indicated (TA and MG). In **(B,C)**, the triceps surae muscle continuously stretched 3 mm **(B)**, 6 mm **(C)**; the stretch was applied approximately 3 s before pinna stimulation. Note in **(B,C)** the shortening of the TA approach period and the pre-extensor discharges; also note in **(C)** the prolonged extensor firing and the interruption of the RP despite continuous pinna stimulation. In **(D–F)**, the lower trace (S) indicates the time of respective electrical stimulation (300 Hz) of the MG filament at 3.1 times the threshold of the most excitable afferent fibers. In **(E)**, the activities commence with extensor activity that terminated although nerve stimulation was maintained. Note in **(F)**, electrical stimulation applied during the RP increased the MG burst duration 7- to 8-fold. The red arrow indicates EMG, ENG burst produces by triceps surae stretching, 3 or 6 mm respectively.

**Figure 13:**
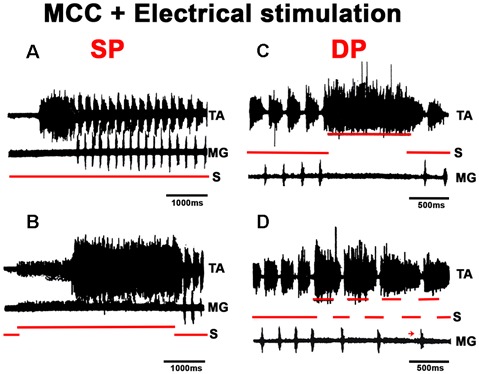
Effects produced by superficial or deep peroneus (DP) nerve electrical stimulation in the FS pattern. **(A,B)** Electrical stimulation to superficial peroneus (SP) prolongs the TA AP. **(C,D)** The DP nerve stimulation prolongs the TA burst with a corresponding decrease in MG activity. When nerve stimulation coincides with TA activity, the duration of TA bursts increases; when the stimulus begins during MG activity, the TA post-discharge also increases but in a minor proportion (arrow).

In MCC, FS began in the TA, 1–5 s after mechanical pinna stimulation. No changes were noted in this pattern after ipsilateral L5–L7 dorsal root sectioning. The duration of the initial TA ENG during the AP ranged from 0.5 to 2.5 s among different animals. The AP period concluded 20–40 ms before the RP onset. The average scratch cycle frequency was 5.5 ± 0.3 Hz, and the durations of the flexor and extensor bursts were 136 ± 35 ms and 17 ± 8 ms, respectively (Figure 11A). MRs in the TA and MG nerves were evaluated to study the MG and TA motoneuron excitability by L7 dorsal root electrical stimulation. The results in Figure 11B illustrate depolarization of the MG before the approach period, which could be subthreshold activity in the MG ENG. MR’s amplitude increased 200 ms before the AP period, and the TA reflex increased several times during the AP. In contrast, the MG reflex decreased before TA activity’s onset and the reflex was depressed (almost abolished). During the RP, TA and MG reflex activity amplitudes varied cyclically from facilitation to depression, almost matching temporarily with the MG or TA’s activity (Figure 11C). Stretching the triceps surae muscle at 3 mm induced a short duration burst in the MG sectioned nerve, and the AP in TA nerve was reduced (Figures 12A,B). When the MG tendon was pulled to 6 mm, the TA approach period duration was reduced even further; RP was also reduced and ceased in association with a prolonged MG ENG discharge (Figure 12C). In two experiments, thin filaments of the MG nerve were sectioned and used for electrical stimulation (30–300 Hz). When the pinna and nerves were stimulated simultaneously, the approach period in the TA nerve did not occur (Figure 12E). Instead, prolonged activity in the MG ENG was observed. When electrical stimulus was applied during the RP, the TA burst duration decreased, whereas the MG ENG duration increased (Figure 12F). This occurred when the stimulus was three to five times the threshold for the more excitable fibers. Electrical stimulation of SP nerves (2 × T, 30–300 Hz) prolonged the AP duration in the TA ENG (Figures 13A,B). Electrical stimulation of the DP nerve during the RP by more than three to four times the threshold of the most excitable fibers also prolonged the TA approach period based on stimulus duration, with concomitant abolishment of extensor ENG (Figure 13C). The extensor’s activity in the MG nerve resumed after a prolonged TA activity. Short duration of electrical stimulus prolonged TA bursts and the MG ENG appeared after the TA post-discharges (Figure 13D). These results indicate that the half-center hypothesis prevails in the RP of MCC.

## Discussion

The presence of scratching rhythmic activity without pinna stimulation occurred in four BCAC. Thus, d-tubocurarine application to C1 affected the rhythmic scratching generator activation. It is important to note that the AP did not appear in these scratching patterns and the RP ranged from 6 to 20 cycles. There was a clear segregation between the AP and RP. Tubocurarine changes the state of the segmental scratching circuit apparatus, particularly, the activity of interneurons and flexor and extensor motoneurons. Flexor associated interneurons that are related to AP were inhibited maybe by a direct action of d-tubocurarine in interneurons with cholinergic receptors, this point deserves further investigation. The short duration of the RP could be attributed to a postural action, where the limbs of the subject are extended (Degtyarenko et al., 1992). The presence of DRPs is indicative of primary afferent depolarization (PAD) in some type of afferent fibers. PAD was not observed in group Ia afferents during scratching (Gossard, 1996; López Ruiz et al., 2017). The presence of DC potentials during the RP could be related to an increase in extracellular potassium (Jiménez et al., 1983) or it also could be produced by electrical coupling between neuronal elements (astroglia or neurons of the scratching generator; Kiehn et al., 2000; Wendy Bautista et al., 2012, 2014). Electrical coupling can generate synchronized oscillations to promote rhythmic firing (Bennett and Zukin, 2004; Connors and Long, 2004); this phenomenon has been described in the mammalian spinal cord (Rash et al., 1996; Kiehn et al., 2000; Lemieux et al., 2010), including among motoneurons in the adult cat (Gogan et al., 1977). DC-induced magnetic fields could probably alter the neuronal excitability by acting in potassium channels (Bączyk and Jankowska, 2018; Kaczmarek and Jankowska, 2018). Magnetic fields have been used to study its effects in motor circuitry activation, such as locomotion initiation with spinal cord stimulation (Avelev et al., 2009); they can also affect cell migration by increasing intracellular calcium and activating signaling pathways (Zhang et al., 2018).

The presence of two rhythms in different and the same motoneuron pools suggests the existence a two-level scratching CPG: one for the rhythm generation and the other for pattern formation (Rybak et al., 2006a,b). It also indicates motoneuron segregation. The slow rhythm (0.5–2 Hz) in SOL motoneurons following FP mechanical stimulation contrasts with the fast rhythm (5–6 Hz) induced by mechanical pinna stimulation. Thus, different pathways converge on a specific set of interneurons for either FS or FL. The activation of silent SOL motoneurons by mechanical FP stimulation in BCAC indicates increased excitability in extensor motoneurons (Power et al., 2010). This is probably mediated by 5HT, which can predominate in specific segregated neurons and produces asymmetrical oscillation. A segregation at the rhythmic level of the spinal cord’s CPG is also possible. However, we did not find alternation between TA flexor motoneurons and MG motoneurons with simultaneous scratching alternating activity in neurons of the same or distal joints.

The descending control activating the rhythm generator could be related to the reticular formation–spinal cord pathway since tonic activity is sufficient to effectively drive motor activity. This includes pacemakers for gait initiation, speed regulation, and their descending targets in the pontine reticular formation of mammals, including humans (Kozlov et al., 2014; Brownstone and Chopek, 2018). In mice, an activation of brainstem interneurons by periventricular glutamatergic neurons that were active by itch and modify the scratching patterns was observed (Gao et al., 2019).

In BCAC, HeMRs produced by PB or MG Ia afferent fibers activate motoneurons innervating muscles acting in different joints. It is not known whether there is a synaptic connection between PB Ia afferents and FHL MG motoneurons. Ephaptic interactions between nerve fibers traversing the lumbar dorsal roots under control conditions and with nerve fiber stimulation have been demonstrated (Bolzoni and Jankowska, 2019). The excitability of these fibers increased coincident with the nerve impulses evoked by a conditioning stimulation. The increase in the excitability lasted 1–2 min (Bolzoni and Jankowska, 2019). We do not know whether there is an ephaptic connection between MG, PB, and FHL Ia afferent fibers. If so, HeMRs could be produced by either MG or PB in FHL motoneurons by FHL Ia afferents ephaptic effects. However, it is interesting that HeMR in distal joints behaves as HeMR in motoneurons of the same joint. This indicates an activation of the scratching generator, which is spread along several segments in the spinal cord (Pérez et al., 2009).

The effects of 5HT on scratching behavior remain unclear. In some experiments, 5HT application to the brainstem produced AP and RP after pinna stimulation, but neither occurred only with pinna stimulation. Thus, neurons with 5HT receptors placed down the obex are elements in the circuit to produce FS by pinna stimulation. This pathway and the circuit should be studied in further experiments. Previous studies have demonstrated the modulation of the locomotor behavior activation by 5HT receptors (5HT7, 5HT2A, and 5HT1A) in the cat spinal cord. 5HT also facilitates neuronal recruitment with the same receptors in neonatal rat spinal cord preparation (Gilmore and Fedirchuk, 2004). In this study, 5HT neuromodulatory mechanisms were described for the first time in cat scratching behavior, particularly during the AP. Increased extensor motoneuron’s excitability through serotoninergic pathways could favor synaptic effects in the extensor motoneurons (Duenas-Jimenez et al., 2017). This 5HT-mediated excitability increase was also seen in studies addressing firing properties, depolarization, hyperpolarization, plateau potentials, and neuronal recruitment during the cat stretch reflex (Hounsgaard et al., 1988). Balanced excitation and inhibition and irregular firing are fundamental motifs in certain spinal network functions (Berg et al., 2007). Therefore, 5HT can modify the FS patterns. Others have reported similar results in turtle and rat spinal cord neurons (Hornby et al., 2002; Gilmore and Fedirchuk, 2004). These points deserve further investigation in cats.

Several computational models based on spinal cord neuronal circuits were developed to explain rhythm and pattern formation during locomotion with two or four limbs (Rybak et al., 2015; Shevtsova et al., 2015; Shevtsova and Rybak, 2016; Ausborn et al., 2018, 2019). Scratching in the cat is performed by one hindlimb, and its pattern consists of a tonic period to move the hindlimb to the scratching area and a rhythmic (5–6 Hz bursting) phase. In many 5HT-treated BCAC, the AP starts with tonic activation of either the extensor and/or flexor. Thus, in the BCAC preparation, the AP initiation appeared without reciprocal inhibition between the extensor’s and flexor’s half-centers. Motoneurons lose rhythmic synaptic drive when some commissural interneurons go silent and others continue to fire rhythmically during ipsilateral flexor deletions (Zhong et al., 2012). In BCAC, the AP with concurrent flexor and extensor activity supports the hypothesis of a CPG organization in which motor patterns are asymmetric and modified by other factors (e.g., subcortical descending pathways or proprioceptive and cutaneous afferents).

In the intact cat spinal cord, extensor motoneurons are inhibited when contralateral group II afferents are activated. After spinal transection, the same stimuli excite these neurons (crossed extension reflex). Funicular lesions and serotonergic fibers (5HT1A receptor agonists) restored the crossed inhibition, and this effect was antagonized by WAY-100135. The tonically active descending serotonergic pathway enables the crossed inhibition and the selection of specific spinal reflex patterns *via* 5HT1A receptor activation *via* the dorsolateral funiculi (Aggelopoulos et al., 1996). In the present study, WAY-100635 produced FS bouts in BCAC and SC. Although the effects of WAY-100635 on 5HT1A receptors and its participation in scratching activity is not clear, it seems to facilitate their generation. The main action is a reduction in TA ENG activity. It could be due to the presence of 5HT1A receptors in this motoneurons pool, in Ia inhibitory neurons or other interneurons implicated in TA scratching patterns. Motor circuit synergy involves several components that adjust motor behaviors such as scratching or locomotion; some have already been described, but others need further investigation (Dougherty, 2005; Talpalar et al., 2011; Cherniak et al., 2014; Boije and Kullander, 2018). The results in this study favor the theory that the AP in BCAC during FS following WAY-100635 treatment is shaped at the pattern formation level.

The FS pattern in MCC remained unchanged after L5-S2 rhizotomy, indicating that the basic scratching pattern depends on the CPG. However, the pathways activated by pinna stimulation and by afferent cutaneous and proprioceptive fibers clearly modify scratching patterns. Stretching the gastrocnemius produces an AP that commenced with extensor activity, followed by flexor tonic activation and a concomitant reduction in the MG’s extensor-motoneurons excitability, as the HeMRs in MG motoneurons are illustrated in Figures 11, 12. This AP pattern may be in accordance with the half-center hypothesis, in which the flexor’s half center inhibits the extensor’s half center. However, it depends on the MG’s Ia afferent fibers acting in the extensor hemicenter of the CPG. The MG’s cyclic MR amplitude can be reduced in part by Ia interneurons. Extracellular recordings from 13 alleged Ia interneurons were obtained and showed that they all fired with the corresponding motoneurons. Intracellular recordings from four of these cells revealed that MG nerve stimulation evoked monosynaptic excitatory post-synaptic potentials. Inhibitory post-synaptic potentials were generated antidromically and had similar amplitude to the hyperpolarizations seen during the RP. The duration of these action potentials was <1 ms at a frequency up to 600 Hz, and they were fired at the end of the silent phase from the TA ENG (not illustrated).

Type Ia interneurons and motoneurons appear to follow a common rhythmical activated interneuron system located in the CPG. Afferent effects in the CPG could be attributed to sensory input; for example, there is evidence of direct access to the rhythm and pattern generation circuits (Frigon and Gossard, 2009, 2010). In this study, the alternating facilitation–depression cycles of the MR, as well as the effects produced by flexor or extensor afferent activation, suggest reciprocal inhibition between half centers. Flexor predominance can be transiently reduced by combining the excitation of extensor muscles afferents with descending activation (Burke et al., 2001). However, it is unclear which afferents or descending fibers produce the observed effects. It will be important to establish what firing patterns occur in rhythm-level neurons to learn how the flexor or extensor phase is stopped and interfere with symmetric alternating activity between the neurons.

## Data Availability Statement

The datasets generated for this study are available on request to the corresponding author.

## Ethics Statement

The animal study was reviewed and approved by The ethical guidelines of the Mexican Official Norm (NOM-062-ZOO-1999) and the National Institute of Health Guide NIH, Publication No. 8023 (1996) for the Care and Use of Laboratory Animals. In addition, the experimental protocols were approved by the Institutional Animal Care and Use Committee (IACUC).

## Author Contributions

The corresponding authors SD-J and LC basically designed the stimulation and recording experimental protocols. SD-J, LC, IA, JL-R, LO-C, and BT-V worked on the standardization and the experimental procedures. SD-J, IA, JD-J, LO-C, RC-A, MT, CT-C, and GM-R were responsible for the data collection, organization and analysis. SD-J, IA, JL-R, LO-C, BT-V, and RC-A organized and realized the figures. Everyone contributed in the manuscript writing.

## Conflict of Interest

The authors declare that the research was conducted in the absence of any commercial or financial relationships that could be construed as a potential conflict of interest.
